# Sexual violence and eclampsia: analysis of data from Demographic and Health Surveys from seven low- and middle-income countries

**DOI:** 10.7189/jogh.09.020434

**Published:** 2019-12

**Authors:** Saverio Bellizzi, Alessandra Nivoli, Paola Salaris, Anna Rita Ronzoni, Giuseppe Pichierri, Francesca Palestra, Ola Wazwaz, Miguel Angel Luque-Fernandez

**Affiliations:** 1 Partnership for Maternal, Newborn & Child Health, Geneva, Switzerland; 2 Department of Neuroscience, Institute of Psychiatry, University of Sassari, Sassari, Italy; 3 Department of Endocrinology, Mater Olbia Hospital, Olbia, Italy; 4 World Health Organization, Cairo, Egypt; 5 Kingston Hospital NHS Foundation Trust, Galsworthy Road, Kingston upon Thames, UK; 6 World Health Organisation, Addis Ababa, Ethiopia; 7 Partnership for Maternal, Newborn & Child Health, Geneva, Switzerland; 8 Department of Non-Communicable Disease Epidemiology, Faculty of Epidemiology and Population Health, London School of Hygiene and Tropical Medicine, London, UK; 9 Department of Epidemiology, Harvard T.H. Chan School of Public Health, Boston, Massachusetts, USA; 10 Biomedical Research Institute of Granada, Non-Communicable and Cancer Epidemiology Group (ibs.Granada), Andalusian School of Public Health, Granada, University of Granada, Spain

## Abstract

**Background:**

Scientific literature has provided clear evidence of the profound impact of sexual violence on women’s health, such as somatic disorders and mental adverse outcomes. However, consequences related to obstetric complications are not yet completely clarified. This study aimed to assess the association of lifetime exposure to intimate partner sexual violence with eclampsia.

**Methods:**

We considered all the seven Demographic and Health Surveys (DHS) that included data on sexual violence and on signs and symptoms suggestive of eclampsia for women of reproductive age (15-49 years). We computed unadjusted and adjusted odds ratios (OR) to evaluate the risk of suggestive eclampsia by ever subjected to sexual violence. A sensitivity analysis was conducted restricting the study population to women who had their last live birth over the 12 months before the interview.

**Results:**

Self-reported experience of sexual violence ranged from 3.7% in Mali to 9.2% in India while prevalence of women reporting signs and symptoms compatible with eclampsia ranged from 14.3% in Afghanistan to 0.7% in the Philippines. Reported sexual violence was associated with a 2-fold increased odd of signs and symptoms suggestive of eclampsia in the pooled analysis. The sensitivity analysis confirmed the strength of the association between sexual violence and eclampsia in Afghanistan and in India.

**Conclusions:**

Women and girls in low-and-middle-income countries are at high risk of sexual violence, which may represent a risk factor for hypertensive obstetric complication. Accurate counseling by health care providers during antenatal care consultations may represent an important opportunity to prevent adverse outcomes during pregnancy.

Violence against women has globally been recognized as a significant human right issue [1] and, as defined by the WHO, a “global health problem of epidemic proportions” [2].

Available estimates indicate that around one-third of women worldwide throughout the world will experience physical and/or sexual violence by a partner or sexual violence by a non-partner [3].

Similarly, around one in 3 women who have been in a relationship report that they have experienced some form of physical and /or sexual violence by their intimate partner in their lifetime [3].

Studies carried in different settings have clearly demonstrated an adverse effect of sexual violence on women’s health [4], which stretches from somatic disorders like chronic pelvic pain [5], to mental adverse outcomes like depression and anxiety [6-8]. Yet, dearth of evidence exists on the impact of experienced sexual violence on labour distress later in life, with very few studies reporting contradictory results on an increased risk of dystocia [9-12]. Another recent report based on prospective data from a Rape Trauma Service (RTS) evidenced an association with obstetric outcomes like antepartum bleeding [13].

Pre-eclampsia occurs in around 2%-8% of all pregnancies [14] and represents one of the major challenges for researchers in terms of etiology and physiological mechanisms; however, the central role of the placenta in its pathogenesis is undisputed [15]. To explain the significant association between intimate partner violence (IPV) during pregnancy and maternal hypertension [16-18], scientists have speculated on behavioral, emotional, and biological mechanisms related to the sexual aggression trauma [19]; more specifically, alterations of specific hormone synthesis and uptake at placental level was claimed to play a key role [18].

We conducted a secondary analysis of national Demographic Health Survey data from seven countries to assess the association between reported lifetime sexual violence and the occurrence of signs and symptoms suggestive of eclampsia around childbirth during the last pregnancy. These data were from Afghanistan (2015), Colombia (2015), India (2005), Mali (2006), Peru (2012), Philippines (2008), Sao Tome and Principe (2008/2009).

Countries under study represent a heterogeneous sample of contexts in relation to the occurrence of sexual violence as well aspects related to the prevention and treatment of preeclampsia/eclampsia.

Afghanistan for instance feature characteristics like conflict and patriarchal culture, especially in rural areas, both associated with very high levels of violence against women [20]. In the recent years there has been increasing recognition that was exacerbates violence against women and girls, with abuses remaining high also in the post-conflict period [21].

On the other hand, Colombia has made substantial progress toward establishing laws protecting women’s right, including the landmark Law 1257, adopted in 2008, which issued regulations to prevent and punish violence and discrimination against women [22]. However, IPV remains a major issue and an estimated 32% of ever-partnered Colombian women aged 13-49 have experienced physical violence from their current or last intimate partner [22]. Colombia had also the second highest 12-month prevalence rate of physical partner violence in a comparative analysis of 12 Latin American countries [22]. Similarly, in Peru violence against women is a widespread issue with about half of women aged between 15 and 49 who have experienced violence from their partners [23].

India shows one of the lowest prevalence (8.5%) of sexual violence in the world; however, this translates into more that 27 million affected [24]. Important to note how only 1% of victims of sexual violence report the crime to the police, perhaps due to the fact that marital rape is not a crime in India and most of the sexual violence episodes occur in marriage [24].

Prevalence of sexual violence is most probably underestimated also in countries like Sao Tome and Principe and Mali, where occurrence is particularly focalized towards students and housewives [25]. Finally, In the Philippines, statistics report that 1 in 20 women and girls age 15-49 have experienced sexual violence in their lifetime [26].

As far as eclampsia is concerned, Afghanistan has one of the highest burdens of maternal mortality in the world, estimated at 789 deaths per 100 000 live births, and hypertensive disorders account for around one fifth of maternal deaths [27]. Detailed analysis of data from 1980 to 2015 has shown no reduction in incidence of eclampsia in India over the last few decades [28]. Finally, a 2017 multicountry analysis has highlighted a prevalence of signs and symptoms compatible with eclampsia as low as 1.2 (95% confidence interval CI = 1.0-1.3) in the Latin American WHO Region (Peru and Colombia) and a proportion as high as 2.5 (95% CI = 2.3-2.7) in the African WHO Region, which included also Mali [29].

## METHODS

### Population, setting and data

DHS are nationally representative random household surveys covering several indicators of population, with particular focus on maternal and child health [30]. All or ever-married women of reproductive age (15-49 years) are the target population in most DHS surveys. DHS guidelines are designed to maximize safety and disclosure, including interviewing only one woman per household, and maintaining complete privacy during the interview [31]. Questionnaire are translated into major local languages and data are collected via face-to-face interviews by trained personnel. In order to maximize the information comparability across countries, the core content for every round of DHS is standard and includes a complete birth and death history for the children of each eligible woman. Additional questions related to pregnancy complications may also be adopted by countries from the survey questionnaire on antenatal, childbirth and postnatal care.

Several countries comprise a specific questionnaire module on exposure to intimate partner violence (IPV), which is measured by binary indicators of physical, sexual, and emotional violence [32]. Information about IPV is collected with no-one else in the household aware that this was done. The violence module is an abbreviated and modified version of the Conflict Tactics Scale [33], which classifies specific acts like “twisting your arm” as physical, emotional, or sexual violence. Ever having experienced any form of violence by their husband or partner and by their most recent husband or partner is respectively obtained from married/cohabiting and formerly married/cohabiting women [32].

Our analysis included only the most recent surveys in these countries presenting data on both IPV and reported life-threatening obstetric complications during birth of the last infant, thus limiting our analysis to one birth per woman.

**Figure 1:**
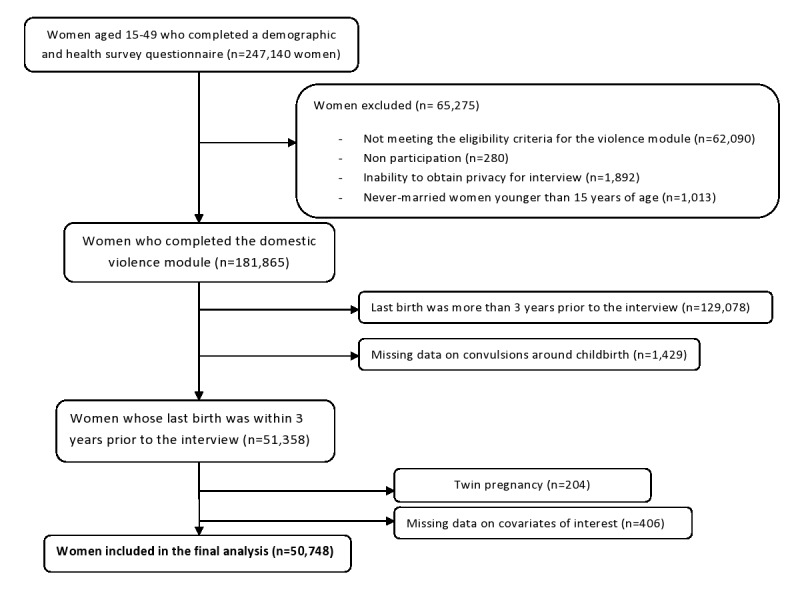
Study participants flowchart.

We pooled all the seven DHS national data sets into one cross-sectional data set containing 247 140 women of reproductive age. We excluded data on girls under the age of 15 and on women not interviewed for domestic violence because of reasons like not meeting eligibility criteria (n = 65 275). For our analysis we only considered the latest pregnancy that occurred within the three years prior to the survey, thus excluding 129 078 women; after excluding records with missing data on convulsions (n = 1429) and on covariates of interest (n = 406) as well as twin pregnancies [34] (n = 204), the final analysis consisted of 50 748 individuals (**Figure 1**).

### Main outcome, exposure and other variables

We used women self-reported occurrence of convulsions not caused by fever as a proxy for the outcome (eclampsia) and women self-reported sexual violence by the partner as the exposure. As indicated by the World Health Organization [35], Intimate Partner Violence (IPV) refers to ongoing or past violence and abuse by an intimate partner or ex-partner – a husband, boyfriend or lover, either current or past. Women may suffer several types of violence by a male partner: physical violence, emotional/psychological abuse, controlling behaviours, and sexual violence.

The index pregnancy corresponds with the closest pregnancy to the DHS interview in case of multiple pregnancies women.

**Figure 2:**
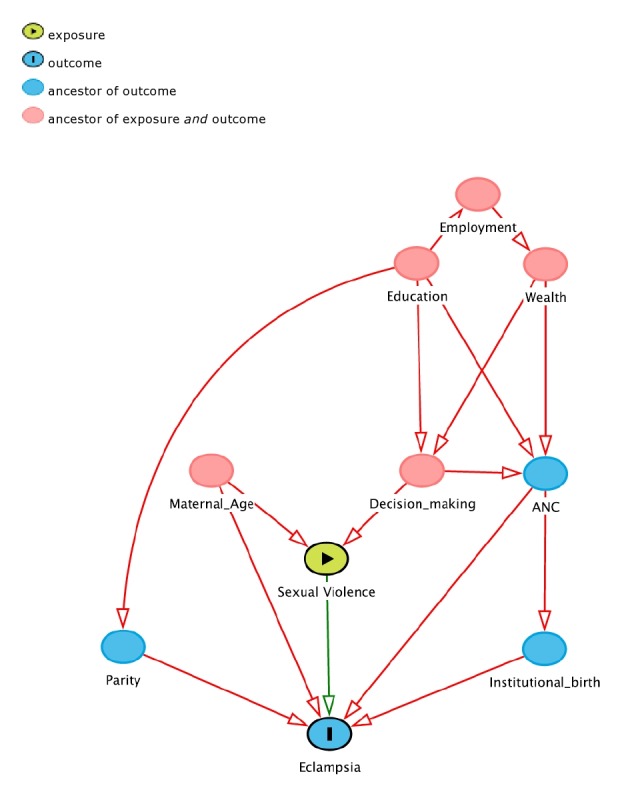
Directed acyclic graph for a proposed causal framework in the association between sexual violence and eclampsia.

We explored several covariates. Maternal age was categorized into three groups, from age 15 to age 24, from age 25 to age 36, and from age 37 to age 49; place of residence was split into urban and rural settings; a wealth index based on asset-ownership and household characteristics data (categorized using the quintiles “poorest”, “poorer”, “middle”, “richer”, and “richest”) was considered as a proxy for socio-economic status [36]. As for literature [37], both maternal and partner’s educational attainment were included after classification in “no education”, “primary”, “secondary”, and “higher”. In consideration of the reported strong association with both maternal hypertensive complications and violence, access to antenatal care and institutional birth as well as self-decision for her own health care (proxy for woman empowerment) were explored [38-40]. We did not consider intendedness of pregnancy as it lies on the causal pathway. Finally, maternal employment status (“employed” and “unemployed”) and birth order categorized in “first birth” and “latter birth” [31] were investigated. We considered all these variables as potential confounders and adjusted for them when assessing the association between eclampsia and sexual violence (**Figure 2**) [38-42].

### Statistical analysis

We use counts and percentages to describe the prevalence of eclampsia and sexual violence by DHS countries. We also described the distribution of reported sexual violence by each covariate.

We evaluated the prevalence of eclampsia and sexual violence across the levels of the covariates in the analysis using crosstabulations and computed the χ^2^ or Fisher exact and trend *P* value tests. Then, to evaluate the risk of suggestive eclampsia by ever subjected to sexual violence we computed unadjusted and adjusted odds ratios (OR) for each country and for the pooled sample. We used a logistic regression. For multivariable analysis, we adjusted for (maternal age, residence, wealth, maternal and partner education, access to ANC and institutional birth, decision making on own health, and parity) and explored the interactions between the occurrence of sexual violence and parity. Considering the lack of information on the exact timing of sexual violence, we conducted a sensitivity analysis restricting our study population to women who had their last live birth over the 12 months before the interview.

Finally, we explored between and within countries heterogeneity for the pooled association between eclampsia and sexual violence and used a nonlinear mixed logistic random effect model to control for the within-country correlation [43].

Confidence interval were calculated using bootstrap technique, based on person-to-person variability (eg, Neyman-Pearson null hypothesis).

We used Stata v13.1 SE (StataCorp LP, College Station, Texas, USA) [44] for statistical analysis.

### Ethical approval

This study used existing data obtained from ORC Macro through formal request mechanisms. No additional ethical review for the secondary analysis was required since each country and the institutional review board of ORC Macro (Calverton, MD, USA) approved the DHS data collection procedures.

## RESULTS

Of the 50 748 women in the seven countries with available data in their Demographic and Health Surveys, the prevalence of self-reported experience of sexual violence was 7.7% (n = 3908), ranging from 3.7% (n = 201) in Mali to 9.2% (n = 1876) in India (**Table 1**). A similar prevalence of women self-reported convulsions with no fever around childbirth (7.9%; n = 4026), ranging from 14.3% (n = 11669) in Afghanistan (the highest prevalence) to 0.7% (n = 2737) in the Philippines (the lowest prevalence) (**Table 1**).

**Table 1:** Prevalence of sexual violence and eclampsia during the last pregnancy in Afghanistan, Colombia, India, Mali, Peru, Philippines, and Sao Tome and Principe, between 2005 and 2015, n = 50 578

Country	Sexual violence		Eclampsia		Total
	**n**	**%**	**n**	**%**	**N**
Afghanistan 2015	997	8.5	1,670	14.3	11 669
Colombia 2015	275	4.9	69	1.2	5 569
India 2005	1876	9.2	2,054	10.1	20 289
Mali 2006	201	3.7	146	2.7	5415
Peru 2012	260	6.2	42	1.0	4200
Philippines 2008	241	8.8	20	0.7	2737
Sao Tome 2008/09	58	6.7	25	2.9	869
Total	3908	7.7	4,026	7.9	50 758

There were no differences in self-reported sexual violence by categories of age. However, it was more common in rural areas. A clear significant linear trend appeared when assessing wealth and education, with the fewer sexual assaults for richest and the higher educated mothers (**Table 2**). A significantly higher prevalence of sexual evidence was present for women with no ANC consultations, no institutional birth, successive birth order position, and for women who could not decide autonomously for their health.

**Table 2:** Descriptive characteristics of interviewees and association with sexual violence in Afghanistan, Colombia, India, Mali, Peru, Philippines, and Sao Tome and Principe, between 2005 and 2015, n = 50 578

Variable		Reporting sexual violence		
	**Total**	**n**	**%**	***P*-value**
**Age in years:**				
15-24	18 290	1414	7.7	0.7
25-36	24 826	1916	7.7	
37-49	7632	578	7.6	
Residence:				
Urban	20 015	1201	6.0	<0.001
Rural	30 723	2707	8.8	
**Wealth quintile:**				
Poorest	11 261	1231	10.9	<0.001
Poorer	11 351	1014	8.9	
Middle	10 569	812	7.7	
Richer	9850	562	5.7	
Richest	7717	289	3.7	
**Education level:**				
None	23 031	2140	9.3	<0.001
Primary	7972	699	8.8	
Secondary	14 803	913	6.2	
Higher	4942	156	3.2	
**Partner’s education:**				
None	15 937	1375	8.6	<0.001
Primary	7686	702	9.1	
Secondary	16 670	1289	7.7	
Higher	4486	220	4.9	
**Employment status:**				
Employed	20 645	1539	8.4	0.08
Unemployed	29 253	2369	8.6	
**ANC:**				
None	10 947	1045	9.5	<0.001
At least one	39 770	2860	7.2	
**Woman empowerment:**				
No	23 594	2039	8.6	<0.001
Yes	27 154	1869	6.9	
**Institutional birth:**				
No	22 732	2191	9.6	<0.001
Yes	28 016	1717	6.1	
**Parity:**				
First birth	12 304	731	5.9	<0.001
Second or more	38 444	3177	8.3	

ANC – antenatal care

**Figure 3:**
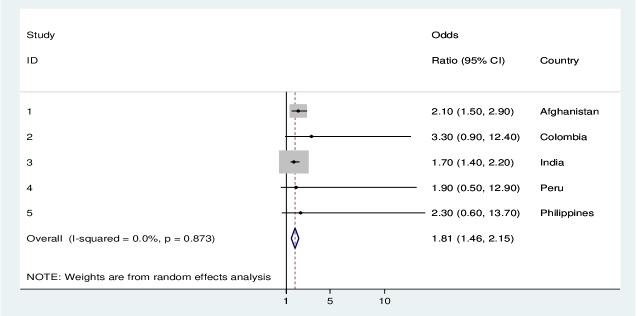
Forest plot showing the association between exposure to sexual violence and signs and symptoms suggestive of eclampsia around childbirth for pregnancies in the previous 12 months, by DHS country (Afghanistan, Colombia, India, Peru, and the Philippines) between 2005 and 2015, n = 19 881.

Reported sexual violence was associated with a 2-fold increased odd of signs and symptoms suggestive of eclampsia in the pooled analysis when adjusted for all the above-mentioned factors. In the individual country analyses, there was a significant association between sexual violence and eclampsia in Afghanistan, Colombia, India, and Mali (**Table 3**, **Figure 3**).

**Table 3:** Association between exposure to sexual violence and signs and symptoms suggestive of eclampsia around childbirth for pregnancies in the previous three years by DHS country (Afghanistan, Colombia, India, Mali, Peru, Philippines, and Sao Tome and Principe), between 2005 and 2015, n = 50 578.

	Eclampsia					
**Country**	**No, n (%)**	**Yes, n (%)**	**cOR**	**95% CI**	**aOR**	**95% CI**
Afghanistan	1495 (89.5)	175 (10.5)	1.3	1.1-1.6	1.6	1.2-2.2
Colombia	63 (91.3)	6 (8.7)	1.8	0.7-4.4	2.3	1.0-5.7
India	1751 (85.2)	303 (14.8)	1.8	1.5-2.3	1.6	1.3-1.9
Mali	128 (87.7)	18 (12.3)	3.9	2.2-6.5	4.5	2.4-8.4
Peru	36 (85.7)	6 (14.3)	2.6	1.1-6.2	2.1	0.9-5.8
Philippines	17 (85.0)	3 (15.0)	1.8	0.4-6.4	1.2	0.3-5.9
Sao Tome and Principe	23 (92.0)	2 (8.0)	1.2	0.3-5.4	1.4	0.6-3.4
All countries	3513 (87.2)	513 (12.8)	1.7	1.3-2.3	2.0	1.5-2.8

cOR – crude odds ratio, aOR – adjusted odds ratio for maternal age, residence, wealth, maternal and partner education, access to antenatal care (ANC) and institutional birth, decision making on own health, and parity, CI – confidence interval

In the subgroup analyses of pooled data for last pregnancy within 12 months prior to the interview, the odds of eclampsia signs and symptoms decreased to 1.8 (95% CI = 1.1-2.8), and remained significant for Afghanistan, Colombia and India, while no data were available for Mali and Sao Tome and Principe (**Table 4**).

**Table 4:** Association between exposure to sexual violence and signs and symptoms suggestive of eclampsia around childbirth for pregnancies in the previous 12 mo by DHS country (Afghanistan, Colombia, India, Peru, and the Philippines) between 2005 and 2015, n = 19 881

	Eclampsia					
**Country**	**No, n (%)**	**Yes, n (%)**	**cOR**	**95% CI**	**aOR**	**95% CI**
Afghanistan	598 (89.7)	69 (10.4)	1.5	1.1-2.0	2.1	1.4-2.9
Colombia	20 (87.0)	3 (13.0)	4.3	1.2-14.8	3.3	0.8-12.5
India	725 (84.2)	136 (15.8)	2.0	1.6-2.4	1.7	1.4-2.4
Peru	13 (86.7)	2 (13.3)	3.2	0.7-14.5	1.9	0.5-12.9
Philippines	10 (76.9)	3 (23.1)	3.4	0.9-12.8	2.3	0.6-13.7
All countries	1427 (87.0)	214 (13.0)	1.5	1.1-1.9	1.8	1.1-2.8

cOR – crude odds ratio, aOR – adjusted odds ratio for maternal age, residence, wealth, maternal and partner education, access to antenatal care (ANC) and institutional birth, decision making on own health, and parity, CI – confidence interval

## DISCUSSION

We found strong evidence supporting the association between the self-reported occurrence of convulsions not caused by fever and women self-reported sexual violence by partner among pregnant women in seven low-and-middle-income countries. Furthermore, this secondary analysis indicated that almost one in ten women in the low-and-middle-income countries under analysis had a history of sexual violence and had their last pregnancy complicated with signs and symptoms compatible with eclampsia. Pooled analysis showed 2-fold higher risk for eclampsia among women who reported lifetime sexual aggression when compared to women with no history of violence after adjusting for all the relevant confounders.

DHS are frequently the only source of maternal health information in low- and middle-income countries [30] and are generally considered of high-quality because of standardized questionnaires and operative procedures [30]. However, the use of cross-sectional data makes it difficult to know whether the sexual violence occurred before pregnancy or vice versa. We attempted to minimize such a problem by restricting the analysis to the last pregnancy taking place within three years prior to the interview; to confirm results, we conducted a sensitivity analysis considering only last pregnancy in the previous 12 months.

Under-reporting violence victimization and perpetration is common in this type of surveys due to recall and reporting bias, which leads to non-disclosure of violent behaviors [4]. The survey methodology was conceived to reduce the reporting bias but could not eliminate it entirely. There is also a possible under-reporting by older women who may have forgotten a violence experienced many years ago. Despite it, a hospital-based study found that recall of convulsions was prone to little inaccuracy (sensitivity = 96.4%; specificity = 87.5%) when compared to the recall of other signs and symptoms of complications, we cannot completely rule out a bias effect [45]. Furthermore, women who experienced violence may be more likely to report adverse pregnancy outcomes [46].

Given that women who experience violence may be more likely to terminate their pregnancy than women who do no, a differential selection bias towards the null may have been introduced [47]. Moreover, for some reasons some women were not interviewed; research showed that women refusing to be interviewed are more likely to have experienced adverse pregnancy outcomes [48].

Also, we could not adjust for unmeasured confounding factors such as alcohol intake or drug use, which have frequently been associated to history of abuse [49].

Violence against women has lately been gaining the deserved attention for the long-term consequences on physical and mental health. Very few analyses reported on the association between sexual aggression and pregnancy complications around childbirth [13,50,51], and to our knowledge, no study explored the effect on maternal hypertensive disorders as a primary objective.

The high variability of eclampsia prevalence across countries has been highlighted in the past. One health facility-based study in particular showed values ranging from less than 1% in Angola up to 8% in Brazil [52]. High prevalence of hypertensive maternal disorders might reflect various aspects, such as variability in maternal risk-factor distribution and poor access to health care services leading to poor management of obstetric complications.

Our results on maternal and pregnancy factors linked to sexual violence are largely confirmed in literature. Woman and partner’s lowest level of education [37], unintendedness of pregnancy [38], and lack of decision making on own health care are all clearly associated with abuse [38,39]. Women who experienced sexual assault generally have inadequate antenatal care [40] and less access to institutional birth [38]. Decision making, here considered as who makes decisions about her own health care, is associated with sexual violence.

A case-control study conducted in Peru found an increased risk of pre-eclampsia for women reporting intimate partner physical and emotional violence (OR = 1.9; 95% CI = 1.1-3.5) during pregnancy [18]. Other two studies investigating the effects of intimate partner violence (IPV) around the time of pregnancy [16,17] confirmed an association with hypertension during pregnancy. On the other side, a prospective cohort including 68 505 women from fourteen US states revealed that women exposed to sexual violence before age 18 were more likely to develop hypertension compared to women with no abuse history [53]. Although pre-eclampsia was not the primary outcome under study, a report found its prevalence higher among women reporting childhood abuse, 5.0% compared with 4.2% among those not reporting childhood abuse (*P* value = 0.013) [53]. Similar results were evidenced in a study investigating on the association between childhood abuse and autism [54].

Abuse before pregnancy may increase the risk of maternal hypertensive disorders through stress, behavioral, or other pathways. Stressful events like sexual violence before pregnancy may lead to conditions like kidney or urinary tract infection, placental problems, gestational diabetes [16], augmented inflammatory markers [55], and autoimmune conditions [56], all conditions related to hypertension during pregnancy [41]. Distress conditions, including intimate partner violence, may directly change the hypothalamic-pituitary-adrenal (HPA) axis, leading to increased cortisol levels [57,58]. Endogenous hypercortisolism as in the case of the Cushing syndrome on its turn, has been found to be associated with hypertensive disorders during pregnancy, including eclampsia [59]. Mechanisms leading to maternal hypertension include alterations such as endothelial dysfunction [60], impaired placental circulaton [61], and altered activity of 11beta-hydroxysteroid dehydrogenase that inactivates cortisol in cortisone [62]. Recent reports focused the attention to the altered expression of cortisol-signaling genes as an important regulatory mechanism related to the epigenetic modification in the placenta and subsequent evolution to pre-eclampsia [63]. Other metabolic alternations like reduced heart rate variability and parasympathetic tone have been implicated in the pathogenesis anxiety disorders/depression, often linked to IPV, and pre-eclampsia [64].

## CONCLUSIONS

The augmented risk of hypertensive obstetric complications reinforces the literature findings on maternal health consequences due to exposure to intimate partner sexual violence. As highlighted by the WHO [65], antenatal care may represent an important opportunity to ask women about violence. This entails enhanced counseling by health care providers when assessing pregnancy-related health complaints, and close monitoring during the third trimester due to higher chances of adverse outcomes.
